# A longitudinal study of gene expression in healthy individuals

**DOI:** 10.1186/1755-8794-2-33

**Published:** 2009-06-07

**Authors:** Chris Karlovich, Guillemette Duchateau-Nguyen, Andrea Johnson, Patricia McLoughlin, Mercidita Navarro, Carole Fleurbaey, Lori Steiner, Michel Tessier, Tracy Nguyen, Monika Wilhelm-Seiler, John P Caulfield

**Affiliations:** 1 Department of Genomics and Oncology, Roche Molecular Systems, Pleasanton, CA, USA; 2 Pharma Development, Molecular Medicine Laboratories, F Hoffmann La Roche Ltd, Basel, Switzerland; 3 Department of Biostatistics and Bioinformatics, Roche Molecular Systems, Pleasanton, CA, USA; 4 Pharmaceutical Development, Methodology and Innovation, Biomathematics, Roche Palo Alto, LLC, Palo Alto, CA, USA; 5 Pharma Development, Clinical, Inflammation Methodology and Innovation, Roche Palo Alto, LLC, Palo Alto, CA, USA

## Abstract

**Background:**

The use of gene expression in venous blood either as a pharmacodynamic marker in clinical trials of drugs or as a diagnostic test requires knowledge of the variability in expression over time in healthy volunteers. Here we defined a normal range of gene expression over 6 months in the blood of four cohorts of healthy men and women who were stratified by age (22–55 years and > 55 years) and gender.

**Methods:**

Eleven immunomodulatory genes likely to play important roles in inflammatory conditions such as rheumatoid arthritis and infection in addition to four genes typically used as reference genes were examined by quantitative reverse transcription-polymerase chain reaction (qRT-PCR), as well as the full genome as represented by Affymetrix HG U133 Plus 2.0 microarrays.

**Results:**

Gene expression levels as assessed by qRT-PCR and microarray were relatively stable over time with ~2% of genes as measured by microarray showing intra-subject differences over time periods longer than one month. Fifteen genes varied by gender. The eleven genes examined by qRT-PCR remained within a limited dynamic range for all individuals. Specifically, for the seven most stably expressed genes (CXCL1, HMOX1, IL1RN, IL1B, IL6R, PTGS2, and TNF), 95% of all samples profiled fell within 1.5–2.5 Ct, the equivalent of a 4- to 6-fold dynamic range. Two subjects who experienced severe adverse events of cancer and anemia, had microarray gene expression profiles that were distinct from normal while subjects who experienced an infection had only slightly elevated levels of inflammatory markers.

**Conclusion:**

This study defines the range and variability of gene expression in healthy men and women over a six-month period. These parameters can be used to estimate the number of subjects needed to observe significant differences from normal gene expression in clinical studies. A set of genes that varied by gender was also identified as were a set of genes with elevated expression in a subject with iron deficiency anemia and another subject being treated for lung cancer.

## Background

Gene expression profiling studies in venous blood are used to explore transcriptional differences between diseased and healthy individuals, identify biomarkers that may identify the appropriate therapy for individual patients, monitor a therapy and pharmacodynamic responses to new drugs, or define prognosis. For example, tumor molecular profiles have been identified that select preferred therapy and predict recurrence [1-3]. For autoimmune diseases, where the disease tissue is not readily accessible, peripheral blood has become a surrogate tissue by default [4]. Venous blood contains cells that will migrate into and/or have migrated from the inflamed tissue, potentially allowing the identification of patients with disease by expression profiling of whole blood. To date, studies of rheumatoid arthritis, systemic lupus erythematosus (SLE), Crohn's disease, and multiple sclerosis (MS) patients have identified expression profiles that may be useful in diagnosis [5,6].

Knowledge of the variability of gene expression over time in healthy men and women will help in the design of studies where drugs are expected to modulate gene expression in the diseased population. It will be particularly important to clearly establish the extent of normal variation for cases where the difference between normal and diseased gene expression profiles is subtle.

Clinical studies need to control for multiple sources of variability since gene expression can be influenced by psychological stress [7,8], exercise [9,10] and eating [11]. In vitro, inflammation markers are rapidly induced in peripheral blood mononuclear cells (PBMC) by infectious agents [12-14]. In vivo gene expression profiles of individuals exposed to a bacterial toxin appeared to be different from those of healthy individuals not exposed to the toxin [15]. Gene expression is also sensitive to the method by which the RNA from blood is isolated [16,17].

Several previous studies have examined gene expression in the peripheral blood of normal individuals [15,18-21]. These investigators found inter-individual variation as well as genes that uniquely identified the subjects being studied. The results were obtained using either peripheral blood mononuclear cells (PBMC) or whole blood. Gene expression profiles differ greatly among the different blood cell types [22,23]. However, the isolation of the different cell types requires capable laboratories that are not always available at all centers involved in large clinical trials. Whole blood can be sampled reliably with the PAXgene Blood RNA System [24,25]. Thus, the studies here were carried out using this collection method.

The gene expression studies mentioned previously were largely limited to a single platform for assays (microarray or qRT-PCR), small numbers of subjects with analyses often restricted to a short sampling time frame, or even a single time point [15,18-21]. This study represents the largest reported study of longitudinal genomic sampling utilizing both microarray and qRT-PCR in an age and gender stratified healthy patient population, to the best of our knowledge. Moreover, the study was performed under controlled conditions with all samples drawn in the morning from fasted individuals at fixed times relative to the start of the study.

The present study extends the information base for healthy individuals by measuring the stability and dynamic range of gene expression in healthy subjects over an extended period of time, six months. qRT-PCR was used to define the range of gene expression in 28 individuals. Eleven immunomodulatory genes likely to play important roles in inflammatory conditions and four reference genes were examined. In addition, 22 subjects were assessed by high-density oligonucleotide microarray, two of whom experienced a severe adverse event during the six-month study period.

## Methods

The study was conducted in accordance with good clinical practice, the Declaration of Helsinki, and appropriate regulatory guidelines. An independent ethics committee approved the protocol. Subjects gave written informed consent to participate in the study.

### Subjects

Blood samples were obtained from 80 healthy volunteers enrolled at a single site in Strasbourg, France. All subjects were enrolled in January and early February, 2003 and had their last visit in August. Subjects were enrolled into one of four cohorts:

• Cohort 1: males 20–55 years of age

• Cohort 2: males > 55 years of age

• Cohort 3: females 20–55 years of age

• Cohort 4: females > 55 years of age

Subjects were enrolled in the study if they were at least 20 years of age and were healthy, as determined by medical history, physical examination, and standard laboratory test values. Exclusion criteria included abnormal laboratory test results, BMI > 32, hypertension, and a positive urine pregnancy test, or positive urine HCV, HBV or HIV test. Any subject who had a concomitant disease or condition that could interfere with clinical evaluations, was an active smoker, or was taking medication on a regular basis was also excluded. Former smoking status was not captured.

A subset of 20 subjects was randomly selected from this study population for microarray analysis. The subset consisted of five subjects from each cohort. Two additional subjects who suffered from serious adverse events during the study were added subsequently. Samples from those 22 subjects were taken at five time points (Baseline, Day 14, Day 28, Day 90 and Day 180).

Quantitative RT-PCR was originally performed on all 80 subjects at each of the five time points for all analytes described in the study. An analysis of the data revealed that mean gene expression in all analytes varied significantly by time point. Due to flaws in the initial experimental design, we could not discern the degree to which the differences were attributable to a true biological effect (e.g. a seasonal effect of gene expression) or experimental bias (e.g. a "batch" effect).

The qPCR study was therefore repeated with 28 subjects chosen at random from the larger set of 80 where sufficient RNA remained. The 28-subject study was designed to distinguish between biological and experimental variability, and to minimize experimental variability. The 28 subjects were assayed by qRT-PCR at four time points (Baseline, Day 28, Day 90 and Day 180). Samples from eight subjects were analyzed on both qRT-PCR and microarray platforms.

### RNA processing and quantitation

Whole blood (2.5 mL) was collected into PAXgene tubes (Becton-Dickinson Diagnostics; Hombrechtikon, Switzerland) and frozen immediately at -20°C. The tubes were shipped frozen and stored at -80°C for a period ranging from one week to six months before RNA was extracted. RNA extractions were performed in batches in the order in which the PAXgene tubes were received. Total RNA was extracted using the PAXgene 96 Blood RNA kit (Qiagen; Hilden, Germany) according to the manufacturer's instructions. RNA quality was assessed on an Agilent Bioanalyzer 2100 (Agilent Technologies; Palo Alto, CA). Any RNA sample with an RNA integrity number (RIN) < 6.5 was rejected and a replacement sample was obtained from a replicate PAXgene tube. The mean yield of RNA from all extractions with RIN ≥ 6.5 was 7.0 μg per tube (1 SD = 2.6 μg).

### Quantitative RT-PCR

Quantitative RT-PCR assays used the fluorescent dye SYBR Green to monitor amplicon formation in a one step format in which the RT step was performed in the same tube as the PCR. All 384-well plates were profiled on an ABI Prism 7900 HT Sequence Detection System (Applied Biosystems Inc.; Foster City, CA). All reactions were performed with a single reagent lot. The cycling parameters were as follows: Step 1: 50°C 2 min for Uracyl-N-Glycosylase (UNG) digest followed by 95°C 1 min to destroy UNG activity; Step 2: 60°C 30 minutes for reverse transcription; Step 3: 50 Cycles of two-step PCR. Each cycle included 95°C 30 sec (denaturation) followed by 60°C 30 sec (re-annealing and extension) Step 4: Slow ramp from 60°C to 95°C to collect data for dissociation curves. UNG was used to eliminate the possibility of carryover contamination.

One well of RNA diluent was run as a negative control for each RNA specimen. RNA diluent consisted of 10 mM TRIS pH 8.0, 0.1 mM EDTA, 0.020 mg/mL poly r(A) RNA (GE Healthcare), and 0.09% sodium azide (w/v). "High input" and "low input" positive controls assessing expression of B2M were run on each plate. The target for the positive controls was human blood peripheral leukocyte total RNA (Clontech) (100 ng for high input and 1 ng for low input). After a run, the plate was inspected to see that Ct values for high input and low input controls fell within a specified range.

Samples were quantified using ribogreen, a nucleic acid stain. After the concentration was determined, the samples were diluted in RNA diluent. 2 ng of RNA were used in a 10 μl single-tube RT-PCR reaction for all assays except IL-6, where 20 ng were used. All reactions were set up in triplicate using a Biomek FX Laboratory Automation Workstation (Beckman Coulter; Fullerton, CA). The primers used for each assay and gene accession numbers are shown in Additional File 1.

### Microarray procedures

Whole genome expression profiles were generated for all samples at scheduled time points (Days 1, 14, 28, 90, and 180) in the subset of 22 subjects with the exception of the Day 180 time point for subject 174 who died prior to the end of the study. A total number of 109 microarrays were hybridized. The Day 1, 90, and 180 samples were obtained in January/February, April/May, and July/August, respectively, and the mRNA was extracted in the order received (see "RNA processing and quantitation"). Additionally, the samples were processed for microarrays in two batches. The first batch was with samples up to Day 28, and the second with samples taken at Day 90 and Day 180. One microgram of total RNA from each blood sample was used to generate biotinylated cRNA, using the Agilent Low RNA Input Linear Amplification procedure (Version 2), with minor modifications (Agilent Technologies GmbH; Waldbronn, Germany); modification: biotinylated UTP and CTP (Enzo Life Sciences; Farmingdale, NY) were used for the in vitro transcription reaction, each at a final concentration of 1 mM. cRNA was purified using Promega SV 96 DNA isolation kit (automated method), as per the manufacturer's instructions (Promega Corporation, Madison, WI) or Qiagen's RNeasy purification kit (manual method) (Qiagen GmbH; Hilden, Germany); performance equivalency of the manual versus automated methods were assessed in terms of yield, quality and reproducibility with control universal human reference RNA and control whole blood RNA (data not shown). The cRNA samples were hybridized overnight to Affymetrix U133 Plus 2.0 full genome oligonucleotide arrays and then stained with Streptavidin-Phycoerythrin according to the manufacturer's instructions (Affymetrix Inc, CA, USA). Arrays were scanned using a GeneChip Scanner 3000 (Affymetrix) and signal intensities were calculated automatically by GeneChip Operating Software (GCOS, version 1.0; Affymetrix). Gene signal intensities were computed using the MAS 5.0 algorithm (component of GCOS 1.0 software). Signal intensities were normalized using a quantile-quantile method [26]. All normalized data were log2-transformed prior to analysis to down-weight the influence of high expression values. The data have been deposited in NCBI's Gene Expression Omnibus (Edgar *et al*., 2002) and are accessible through GEO Series accession number GSE16028 .

### Statistical analysis

#### qRT-PCR

Units of cycle threshold (C_t_), were used for the expression data for all analyses. Outliers were defined as data points falling ≥ 3 standard deviations (SD) outside the mean C_t _value, averaged over all time points and individuals, within a gene. A graphical assessment was performed to look for possible seasonal effects.

In addition to the normalization to total RNA input that was performed, a strategy of normalization to four reference genes as a function of average gene expression weighted by their variances was considered. Several aspects of our study led us to conclude that reference gene normalization was introducing several sources of systematic variability into the data. This variability was estimated to be of appreciable size (analyses not shown) and was not present in the total RNA normalized data because it had been controlled for through study design. This variability was due to differences among reactions and differences across plates. The differences among reactions were due to the necessity of having reference genes measured in external reactions, because SYBR green assays cannot be multiplexed. The differences among plates were due to our 28-subject substudy design where a single gene was assayed per plate, which allowed us to make all analyses on within-plate comparisons only. It is known that the precision that can be achieved with reference gene normalization is limited by the variability present in the reference genes [27]. Since we could not increase the precision of the immunomodulatory gene expression data with the reference genes, and we had alternatively controlled for appreciable amounts of systematic variability through study design, the strategy of normalization to reference genes was abandoned.

A linear mixed model with fixed effects for gender, the interaction of gender and time, and age, and a random effect for subject was applied to the qRT-PCR data. From this model, effects were tested and within-individual variation in gene expression over time was estimated. The analysis was implemented using SAS Proc Mixed.

#### Microarray

An exploratory analysis was first performed to assess outliers and to determine if any clusters of subjects could be observed. For this purpose, a correspondence analysis was used. This analysis reduces the complexity of the data and facilitates their interpretation by finding combinations of genes that best explain the variability in the entire data set [28]. The analysis was performed using the statistical package XlStat 6.0 (Addinsoft; New York, NY).

### Identification of differentially expressed genes

Of the potential 54,675 probe sets on the U133 Plus 2.0 microarray, 34,573 probe sets were present in at least one sample (among the 109 microarrays hybridized) and were used for the analysis. A statistical analysis was performed to determine if expression profiles could indicate significant gender, age, and time effects on gene expression. A linear mixed-effect model [29] was built independently for each of the 34,573 selected probe sets using log_2_-transformed expression values and the analysis was implemented using the function *lme *in SPLUS.

The model used can be expressed as:



where *y *is the normalized signal intensity of probe set *p *in individual *i *at time *t*, β_*gender*_, β_*age*_, and β_*time *_are respectively the gender, age and time effects and α is the individual random effect. The variances were denoted  (between-subjects variance) for  and  (within-subject variance) for *ε*_*itp*_.

The significance of the effects was tested with *t*-tests. Because of the numerous t-tests performed, we needed to use a correction procedure, called the False Discovery Rate (FDR) [30], which controls the expected proportion of genes erroneously identified as differentially expressed genes. For this study, an FDR of 5% was chosen. For each probe set, a Shapiro test was performed to check whether the linear mixed-effect models correctly fit the data. Only 17,329 probe sets for which the Shapiro P-value > 0.1% were kept for further analysis.

Using the variance components estimated with the linear mixed-effect model built for each of the 17, 329 probe sets, we determined for each probe set *p *the intraclass correlation coefficient among expression measures within a subject over time by calculating the term , with  the variance computed between subjects, and  the within-subject variance. Values of the correlation range between 1 (i.e. no variation within subject) and 0 (i.e. high variation within subject).

We used the graphical representation proposed by Bland and Altman [31] to determine the level of agreement between gene expression values measured at different time points. This type of graph plots the difference between two measures as a function of their means. A good agreement between different measures is observed when the cloud of data points is located around 0 and from the absence of a trend (i.e., slope of a robust linear fit of the scatter plot close to 0). In the graphs shown the differences on the vertical axis and the means on the horizontal axis were not computed from the normalized expression values but from the effects determined by the linear mixed-effect model using time as a factor.

### Infections

Subjects who experienced an adverse event had an additional sample collected following recovery from their infections. Eighteen of the eighty subjects in the study had a total of 24 infections. Infections in 7 patients were ongoing at the time of a scheduled sample collection, and infections in 11 patients ended within fourteen days before a collection. The other seven infections in these patients occurred outside these time frames and were not included in this analysis. There was a wide range of times between the scheduled blood draw and the recovery blood draw (9–51 days), with most recovery period blood draws occurring between 9–22 days. The time between the end of the infection and the recovery period blood draw ranged from 14–97 days, with most occurring between 14 to 23 days after the end of the event.

## Results and Discussion

An overview of the study population and hematology measures is presented first, followed by gene expression data from both individuals who remained healthy throughout the study, and those who experienced an adverse event.

### Study population characteristics

The healthy volunteers, 79 Caucasians and 1 Oriental, were from eastern France and stratified into four cohorts of 20 individuals each based on age (22 – 55 years and > 55 years) and gender (Table 1). The average age of males versus females in each of the two age groups was similar: 34.5 ± 7.8 (mean ± SD) years for males versus 32.3 ± 9.5 years for females in the age 20 – 55 years group, and 59.7 ± 3.1 versus 59.5 ± 4.5, respectively, in the over 55 group. Patients were excluded from the trial if they took medication on a regular basis including birth control, hormone replacement therapy, or herbal medicines or used tobacco. The most frequently used medication for adverse events that occurred during the study was paracetamol (30 subjects), followed by hexetedine (4), phloroglucinol (4), and amoxicylline (4). One subject died from lung cancer prior to the end of the study, while all other subjects completed the five scheduled visits on Days 1, 14, 28, 90, and 180 per protocol.

**Table 1:** Counts of patient samples by study, gender and age group.

**Gender**	**Age Group**	**Main Study (n = 80)**	**qRT-PCR Study (n = 28)**	**Microarray Study (n = 22)**
Female	≤ 55 Years	20	8	5

	> 55 Years	20	7	7

Male	≤ 55 Years	20	5	5

	> 55 Years	20	8	5

A subset of 28 individuals was chosen from the larger study population and analyzed for the expression of selected genes by qRT-PCR at four time points (Baseline, Day 28, Day 90 and Day 180) (Table 1). Gene expression of twenty-two subjects was assessed by high-density microarray. Samples from eight subjects were analyzed on both qRT-PCR and microarray platforms. All other samples in the two subsets were assayed on only one platform.

### Hematology

Complete blood cell counts were obtained from all 80 subjects at all five time points. Neutrophils and lymphocytes, which together account for ~90% of white blood cells, had similar distributions (Table 2 and Additional File 2). Most subjects had cell counts that were in the lower half of the normal laboratory range for each cell type. The within subject estimate of variability for neutrophils was 21% of the mean, and was 16% for lymphocytes. The ratio of the top to the bottom of the range of values measured in the study was 6.3 for neutrophils and 5.7 for lymphocytes.

**Table 2:** Hematology values of all subjects over all time points.

**Cell Type**	**Mean ± SD (10^9^/L)**	**Study Range (10^9^/L)**	**Lab Normal Range (10^9^/L)**	**Within-Subject Estimate of Variability (10^9^/L)**
Total WBC	6.25 ± 1.43	3.3–12.3	4.5–10	0.84

Neutrophil	3.38 ± 1.07	1.4–8.8	1.8–7.5	0.70

Lymphocyte	2.16 ± 0.63	1.0–5.7	1.0–4.0	0.34

Monocyte	0.48 ± 0.13	0.03–1.09	0.20–1.00	0.09

Basophil	0.03 ± 0.02	0.01–0.16	0.00–0.20	0.02

Eosinophil	0.19 ± 0.12	0.04–0.69	0.04–0.50	0.05

Platelet	242 ± 54	129–596	150–400	23.11

The within-subject estimate of variability is the square root of the estimated residual variance from a linear mixed effects model with fixed effects for age, gender and time, and a random effect for subject.

### Range of expression in selected immunomodulatory and reference genes by qRT-PCR

**Figure 1:**
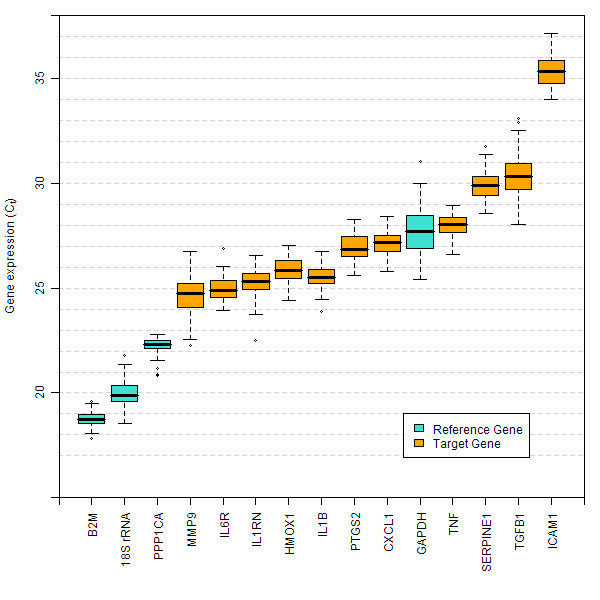
**Total ranges of immunomodulatory and reference gene expression (qRT-PCR study)**. The total range of gene expression is represented by box and whisker plots for immunomodulatory genes (blue) and reference genes (gold), for 28 subjects at four time points. For each gene, the median is indicated by a horizontal line, the boxes indicate the range between the 25th to the 75th percentile, the whiskers indicate the range containing 1.5 times the interquartile range, and circles above the whiskers indicate outliers. Values beyond the range included by the whiskers are indicated as individual points. For ICAM1, three measurements were below the limit of quantification (C_t _> 37).

**Figure 2:**
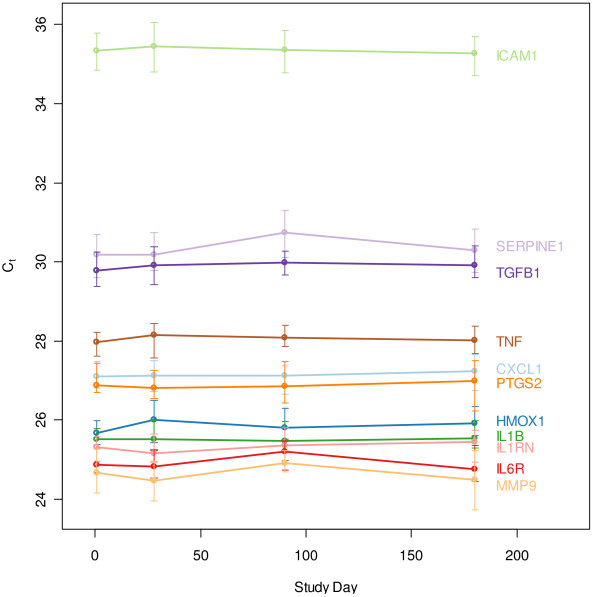
**Immunomodulatory gene expression over time (qRT-PCR study)**. Median gene expression for each immunomodulatory gene is shown, with bars indicating the range from the 25^th ^to the 75^th ^percentile for all subjects.

The RNA expression of eleven inflammatory markers (CXCL1, HMOX1, ICAM1, IL1B, IL1RN, IL6R, MMP9, PTGS2, SERPINE1, TGFB1, TNF), and four genes commonly used as reference genes in blood [32,33] (B2M, 18S rRNA, GAPDH and PPP1CA), was evaluated by qRT-PCR (Figure 1). The dynamic range of expression for all the genes, both inflammatory and reference, was relatively narrow. More specifically, 95% of all samples profiled for CXCL1, HMOX1, IL1B, IL1RN, IL6R, PTGS2 and TNF had a range of gene expression that fell within 1.5–2.5 C_t_, an approximately 4–6 fold range in transcript number, similar to the range for neutrophil and lymphocyte cell numbers. The gene that exhibited the highest variability was TGFB1, in which 95% of all samples spanned 4.8 C_t_, or a 28-fold range. Shapiro-Wilkes tests and Quantile-Quantile analysis demonstrated that expression was approximately log-normally distributed for most targets, with slight deviations in the tails (data not shown). Median expression values for each immunomodulatory gene evaluated remained stable across each of the four time points (Figure 2). Each qRT-PCR assay was performed using an equivalent amount of RNA from all samples as input. Normalizing against any of the four reference genes was not effective at increasing precision of the observed inflammatory gene expression (see Additional File 3).

McLoughlin et al.[15] evaluated expression of 48 inflammation and immune-related transcripts by qRT-PCR in healthy individuals at a single time point, including 10 of the genes in the present study. They found a similarly narrow range of expression between subjects, including standard deviation values that were within 0.2 C_t _of that observed here for most targets examined. Two of the targets with the highest variability in our study, MMP9 and SERPINE1, showed similarly higher variability in that study.

While PPP1CA and B2M had a relatively narrow range of expression, GAPDH and 18S rRNA were more variable among individuals than several of the immunomodulatory genes and therefore were not suitable for normalizing the data set. GAPDH is elevated in T cells upon activation [32,34] and can fluctuate in response to the changing energy demands of cells [35]. The results presented here support the notion that normalizing qRT-PCR data by input RNA amount is a reasonable approach and may introduce less variability than the use of a single reference gene.

### Variation in gene expression over time in samples hybridized on microarrays

**Figure 3:**
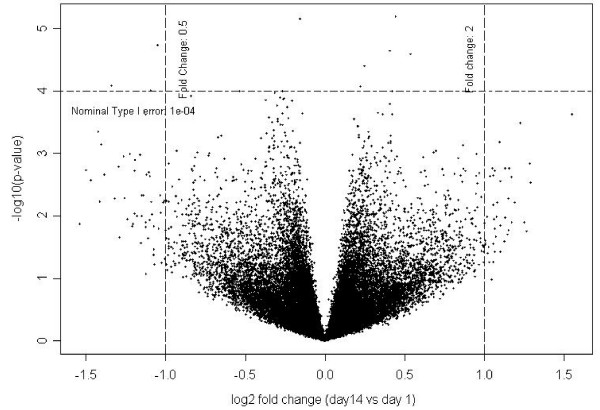
**Time effect of Day 14 versus Day 1 (Microarray study)**. Each cross represents a probe set. The fold change is displayed on the *x*-axis (in log_2 _units); the *p*-values (corresponding to the t-tests performed) are displayed on the *y*-axis (in log_10 _units). Fold changes and *p*-values were computed with the linear mixed-effect model described in the Methods section. The left and right vertical lines represent fold changes equal to 0.5 and 2, respectively. The horizontal line represents an arbitrary type I error equal to 10^-4^.

**Figure 4:**
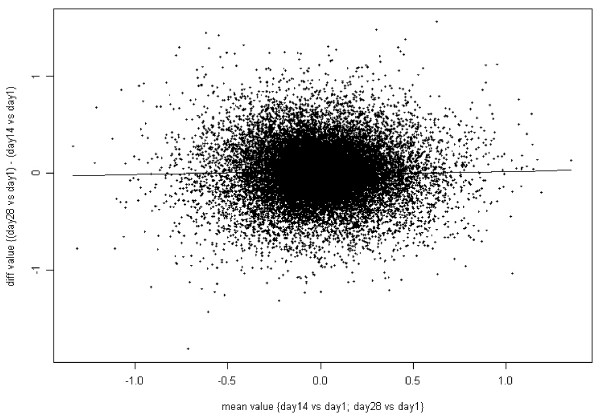
**Effect of time on gene expression (Microarray study)**. **A. Time effect within one month: Day 14 vs. Day 28**. Each dot corresponds to a probe set. The differences on the vertical axis and the means on the horizontal axis were not computed from the normalized expression values but from the effects (*β*_time, p_) determined by the linear model (see Methods), time being either Day 14 or Day 28; Day 1 was defined as baseline. The model used log2 values for the gene signal intensities. A robust local linear fit of the data is represented as a black line.

Pairwise comparisons were performed to determine whether the mean expression of each probe set on the array varied between any two time points. None of the differences seen at Days 14 and 28 compared to Day 1 remained significant after correcting for multiple comparisons (Figure 3). The Bland-Altman plot [31] (Figure 4) shows there is no difference for samples separated by two weeks (e.g. when Day 14 is compared to Day 1 or when Day 28 is compared to Day 14). However, when Day 90 was compared to Day 180, 248 probe sets were found to be differentially expressed, a higher number than would be expected by random chance. Those 248 probe sets correspond to 157 unique genes (excluding probe sets which are not correctly mapped to the genome). Among those 157 genes, 66 genes were found associated with apoptotic activities with log2-fold change ranging from -0.9 to 0.6. It was not possible to draw a conclusion from the comparison of the gene signal intensities measured at Day 1 and at Day 90 (and at Day 1 and Day 180) as samples were processed separately in two batches. The differences we found may be the result of a seasonal effect, a true biological effect, or experimental bias. The observed stability of the gene expression profiles over a one-month period is consistent with results from other studies [19,21].

### Within-individual variability in gene expression over time

#### qRT-PCR

The estimate of total within-individual variance of the eleven inflammatory genes shown in Table 3 reflects the amount of deviation around the mean trend of gene expression for a target gene over time. Total within-individual variance estimates for the eleven inflammatory genes ranged from 0.220 to 0.838 C_t_^2^, which was 1–3% of the mean gene expression for a given gene. The two genes with the highest within-individual variance estimates were MMP9 and SERPINE1, while TNF had the smallest. From the mixed effects models for each mRNA measured by qRT-PCR, it was estimated that the correlation of gene expression among time points in a single individual is less than 50% for all genes assayed.

**Table 3:** Estimates of different components of within-individual variation (qRT-PCR study).

**Gene**	**Variance Within Time Point (C_t_^2^)**	**Covariance Between Time Points (C_t_^2^)**	**Total Within-Individual Variance (C_t_^2^)**	**Correlation Between Time Points**
CXCL1	0.155	0.108	0.263	0.410

HMOX1	0.150	0.097	0.247	0.393

ICAM1	0.512	0.064	0.576	0.111

IL1B	0.191	0.125	0.315	0.395

IL1RN	0.224	0.135	0.359	0.375

IL6R	0.292	-0.018	0.274	-0.066

MMP9	0.612	0.011	0.622	0.017

PTGS2	0.215	0.104	0.319	0.325

SERPINE1	0.503	0.336	0.838	0.401

TGFB1	0.259	0.084	0.344	0.245

TNF	0.150	0.070	0.220	0.318

#### Microarray

**Figure 5:**
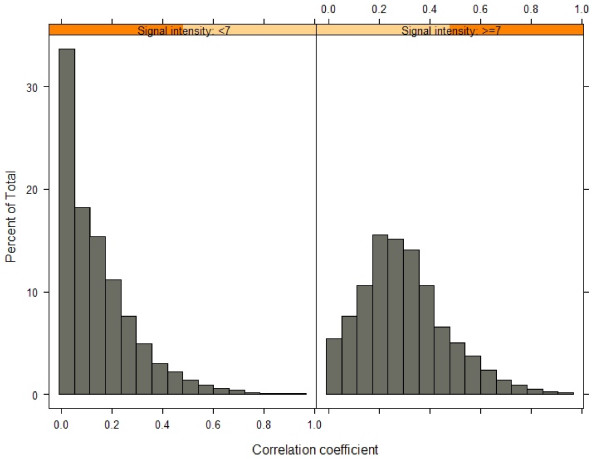
**Distribution of correlation coefficients among probe sets (Microarray study)**. Probe sets satisfying the Shapiro test were divided in two sets according to their average signal intensities: below or above log_2 _7. Distributions of the probe sets according to correlation coefficient values are displayed for both sets.

One measure of the temporal variability of a gene is how well its expression is correlated in any individual from one point in time to the next. Using the variance components estimated with the linear mixed effect models, we determined the correlation coefficient among expression measures within a subject over time for each of the 17,329 probe sets satisfying the Shapiro test (see Methods). Only 10% of the probe sets exhibited a coefficient > 0.4 (Figure 5). However, when only probe sets demonstrating robust transcriptional expression with a log_2_-transformed signal intensity ≥ 7 (raw signal intensity > 100) were considered, the percentage with an intraclass correlation coefficient > 0.4 increased to 23%. The observation that those genes expressed at low levels had the lowest intra-individual correlations may be partly due to the known characteristic of probe sets at the lower end of the quantitative range of a microarray to have poorer reproducibility [36]. For example, Dobbin et al. [37] found that RNA profiles from tumor tissues had weaker correlations within an individual for those genes with low levels of expression. Inspection of the probe sets with the lowest correlation coefficient (< 0.1) but still with a log_2 _expression signal intensity ≥ 10 showed that ~90% either do not map to a unique gene locus or do not map to the current version of the human genome, as already observed by Zhang [38].

### Age and gender effects

No genes analyzed by either qRT-PCR or microarray were observed to be differentially regulated between the two age groups. An age effect has been observed previously with RNA profiling studies [19,21,39], and might have been expected here, given the large number of genes measured. One explanation is that the broad age range within a given group in this study may have prevented the detection of such an effect. Another is that blood might be less susceptible to senescence effects when compared to other tissues such as brain or muscle [40,41].

Females showed a small but statistically significant 1.3–1.5-fold increase in gene expression relative to males for the qRT-PCR targets CXCL1, HMOX1 and ICAM1 (see Additional File 4. On the microarray, a total of 78 unique genes were found to be differentially regulated by gender. As expected, the XIST gene was found to be highly down-regulated in males compared to females, with an average log_2_-transformed signal intensity equal to 50 in females and roughly 20 times lower expression in males. Fifteen of the 78 genes had a log_2_-transformed signal intensity ≥ 7.0 (Table 4). For all of these genes, the mean difference between sexes was less than 20%. Four X chromosome genes were identified as differentially expressed. One of these, RPS4X, is known to escape X inactivation [42] while another, E1F1AX, has family members known to escape X inactivation. Only 23 out of the 78 probe sets mapping on the Y chromosome were present in at least one sample and their log2-transformed signal intensities were always below 7. The majority of the highly expressed probe sets that were differentially regulated by gender are located on autosomal chromosomes (Table 4). Among those genes, flotillin1 (FLOT1), which was down-regulated in males compared to females, was recently identified as an estrogen-responsive gene [43].

**Table 4:** Genes differentially expressed between genders with log-transformed signal intensities ≥ 7 (Microarray study).

**Gene Name**	**Chromo-some**	**Locus ID**	**Affy ID **	**Fold Change**	**Description**
-	-	-	211074_at	0.82	Homo sapiens non-functional folate binding protein mrna, complete cds
EIF1AX	X	1964	201019_s_at	0.86	Eukaryotic translation initiation factor 1a, x-linked
TMEFF2	M	23671	224321_at	0.87	Transmembrane protein with egf-like and two follistatin-like domains 2
FLOT1	6	10211	210142_x_at	0.87	Flotillin 1
EIF2S3	X	1968	224936_at	0.90	Eukaryotic translation initiation factor 2, subunit 3 gamma, 52 kda
RPS4X	X	6191	213347_x_at	0.91	Ribosomal protein s4, x-linked
MGC71993	17	440400	224573_at	0.93	Similar to dna segment, chr 11, brigham + womens genetics 0434 expressed
EEF1A1	1	1915	213477_x_at	1.05	Eukaryotic translation elongation factor 1 alpha 1
EEF1A1	6	1915	206559_x_at	1.07	Eukaryotic translation elongation factor 1 alpha 1
SPOP	17	8405	204640_s_at	1.07	Speckle-type poz protein
ERBB2IP	5	55914	217941_s_at	1.09	Erbb2 interacting protein
UHMK1	1	127933	224691_at	1.11	Kinase interacting with leukemia-associated gene (stathmin)
PP784	4	114932	212199_at	1.12	pp784 protein
HMGN4	6	10473	209787_s_at	1.13	High mobility group nucleosomal binding domain 4
C10orf45	10	83641	223058_at	1.13	Chromosome 10 open reading frame 45
HTATSF1	X	27336	202602_s_at	1.14	HIV tat specific factor 1
GNG2	14	54331	224964_s_at	1.14	Guanine nucleotide binding protein (g protein), gamma 2
HMGN4	6	10473	209786_at	1.17	High mobility group nucleosomal binding domain 4
HMGN4	6	10473	202579_x_at	1.20	High mobility group nucleosomal binding domain 4

XIST gene expression was found to be highly down-regulated in males compared to females. XIST does not appear in this table because the mean signal intensity was below the threshold of log_2_7.

### Effect of two severe adverse events on gene expression

**Figure 6:**
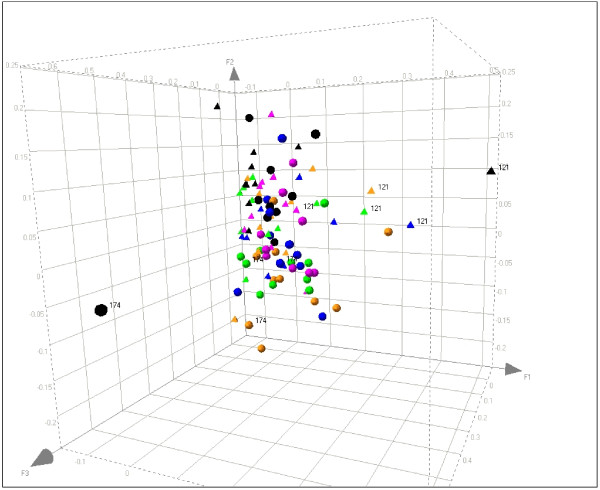
**Correspondence analysis of microarray samples**. Correspondence analysis was performed on gene intensity signals measured on a subset of 22 individuals. Circles represent females; triangles represent males. Time points are colored as follows: orange = Day 1; green = Day 14; blue = Day 28; black = Day 90; pink = Day 180. Outliers are numbered (Subjects 121 and 174). Subject 174 died prior to Day 180; therefore, that sample was not available for analysis. The three axes displayed explain 32% of the variance of the whole dataset. Each data point on the graph represents a projection of the expression profile (34,573 probe sets in this case; see materials and methods for filtering of the data set) of one subject at a single time point. The distance between subjects reflects the distance between their entire gene expression profiles.

A correspondence analysis (see Methods) was performed on a set of 34,573 probe sets for all the samples from the 22 individuals studied with microarrays. This analysis looked for combinations of genes that would explain the overall variability in the data set and identify possible outliers. Almost all individuals across all time points were in close proximity to one another (Figure 6). However, data points from two subjects, numbered 121 and 174, were outside of the main cluster.

Subject 121 had anemia secondary to rectal bleeding with a progressive drop in red cell count from baseline to Day 90. The subject's bleeding was stopped surgically on Day 100; on Day 180 red cell parameters had returned to the normal range. The correspondence analysis data points on Days 1, 14, and 28 are outside of the main cluster with the Day 90 profile reaching a maximum distance from the others. The Day 180 point has returned to normal. RBC-associated genes with elevated expression levels included ferrochetalase, carbonic anhydrase, ALAS2, erythrocyte membrane protein band 4.2, glycophorin A and B, and 2,3-bisphosphoglycerate mutase. Some of the genes over-expressed in subject 121, were correlated previously to reticulocyte expressed genes: SLC4A1, EPP42, BCL2L1 and BNIp3L [21]. ALAS2 and carbonic anhydrase have also been identified as genes that are down-regulated in anemia during acute renal allograft rejection [44].

Subject 174 developed lung cancer and began chemotherapy on approximately Day 70. She was a strong outlier in the correspondence analysis on Day 90. Her monocyte count on that day was severely depressed to 6.5% of the baseline value (from 0.46 × 10^9^/L to 0.03 × 10^9^/L). All other white cell types were within normal limits on Days 1, 14, 28, and 90. For subject 174, 31 genes were found to be up-regulated. The gene ACRBP was elevated four- fold above the other subjects on Day 90. ACRBP is a member of the cancer/testis family of antigens, is immunogenic, and has been detected in different tumor types [45]. The growth arrest-specific (GAS)2-like 1 gene, an actin-associated protein expressed at high levels in growth-arrested cells [46], was found to be 1.5-fold higher in subject 174, compared to other individuals at Day 90. The changes in gene expression observed in this subject correlated with the onset of chemotherapy and may be largely attributable to treatment. However, for both these cases data is required in larger numbers of patients to draw firm conclusions.

### Effect of infection on gene expression

**Figure 7:**
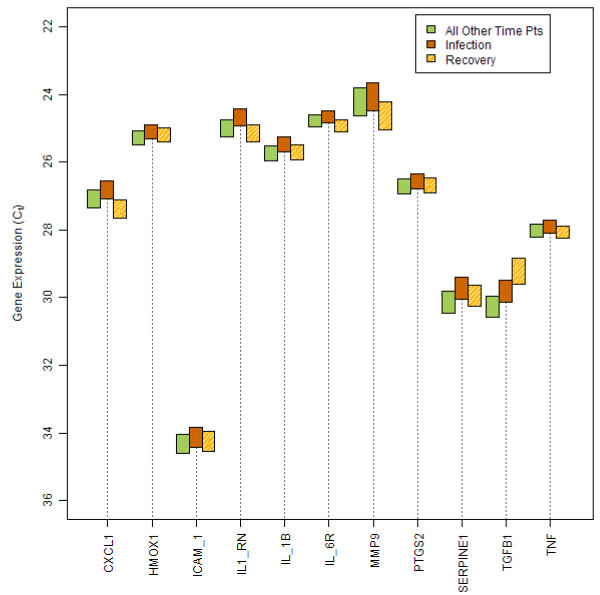
**Comparison of 95% confidence intervals of mRNA levels during an infection, at recovery, and at all other time points (qRT-PCR study)**. A comparison of mRNA levels (in terms of C_t _units) for 12 genes in 18 subjects who experienced an infection during the course of the study. Confidence intervals were obtained from estimates of mRNA levels during the three events using a mixed model that accounted for intra-individual correlation between observations. The y-axis has been reversed to reflect that mRNA levels are higher for lower values of C_t_.

Eighteen of the eighty subjects in the study had a total of 24 infections. The infections were predominantly upper respiratory infections (sinusitis, rhinitis, nasopharyngitis, or pharyngitis), in addition to gastroenteritis, bronchitis, and skin infection and oral herpes simplex. Immunomodulatory gene expression in subjects with active infection at the time of sampling or within two weeks were slightly elevated relative to the expression levels observed two weeks after recovery and at all other time points (Figure 7). However, the expression of TGFB1 was still increased in samples taken after recovery.

## Conclusion

In this study with 80 healthy male and female subjects ≥ 20 years of age, a normal range of gene expression was established for 11 inflammation-related genes and four housekeeping genes. Each gene was stably expressed, independent of age and gender. There was no apparent correlation between values more than three standard deviations from the mean with adverse events or hematology values. Nineteen subjects who had infections at the time of or within two weeks before their scheduled blood draws gave an additional sample two weeks after the resolution of their infections. The samples collected during the active infection showed slight elevations from the expression levels seen in uninfected subjects. However, the samples taken two weeks later showed increased expression of TGFβ1.

Gene expression levels, as measured by microarray analysis, appeared to be constant over one month; however, over three months, a small percentage of genes appeared to vary. We observed that intra-individual gene correlations differ greatly depending on signal intensity. A small proportion of genes were found to be differentially regulated according to gender. Differential gene regulation by age (in subjects 25–55 years of age versus subjects > 55 years of age) was not observed. Elevated expression levels of red blood cell-associated genes were observed in one subject who experienced progressive anemia secondary to blood loss. Included among those genes were ferrochetalase, carbonic anhydrase, ALAS2, erythrocyte membrane protein band 4.2, glycophorin A and B, and 2,3-bisphosphoglycerate mutase.

### Use of RNA expression studies in clinical trials

Several lessons can be drawn from this study regarding the design and conduct of future clinical trials that test new pharmacological entities and employ gene expression as an assessment:

1. Estimates of sample sizes required to achieve a certain power to detect changes in gene expression of a particular magnitude can be calculated from the estimates of variance components given in the results section, results from other studies, and the estimated effect size from *in vitro *work.

2. Samples should be analyzed at the end of the experiment rather than on an ongoing basis. A bias was introduced when samples from the present study were processed and analyzed with different reagent lots over time in sequential batches This type of bias, also recently described by Yang et al. [47], could lead to the identification of many artifacts among differentially expressed gene sets.

3. Small elevations in inflammatory gene expression produced during upper respiratory infections generally return to normal levels of expression within two weeks after the infection has resolved.

4. Time and/or seasonal effects may be a factor in trials lasting longer than one month.

5. Gender and age effects are not likely to be problematic in populations from 20 to 65 years of age. The assessment of gene expression profiles in children or very old individuals might lead to different conclusions.

## Competing interests

This study was funded by Roche and sponsored by Roche Palo Alto.

## Authors' contributions

CK processed and quantified RNA from PAXgene tubes; developed assays, performed experiments and analyzed data for qRT-PCR, and drafted part of the manuscript. PM performed the microarray experiments, analyzed data and drafted part of the manuscript. CF, MT and MWS performed the microarray experiments. AJ performed statistical analyses on the qRT-PCR data and drafted part of the manuscript. GD performed statistical analyses on the microarray data and drafted part of the manuscript. MN performed statistical analyses on the adverse events and hematology data. LS and TN performed experiments for qRT-PCR. JC conceived of the study and its experimental design, participated in the interpretation of results, and helped prepare the manuscript.

## Pre-publication history

The pre-publication history for this paper can be accessed here:



## Supplementary Material

Additional file 1 **Primers used for qRT-PCR**. The table provided describes the primer sequences used for qRT-PCR. Click here for file

Additional file 2 **Frequency distributions for white blood cell types for all subjects, all post-enrollment values**. The histograms show distributions of cell counts for the major white blood cell types. Click here for file

Additional file 3 **Variability of raw and normalized Ct values (qRT-PCR study)**. The table shows the intra-individual variability in qRT-PCR Ct values for the subjects in the study. Click here for file

Additional file 4 **Immunomodulatory genes differentially expressed by gender or age group (qRT-PCR study)**. The table shows variability in qRT-PCR Ct values between genders and subjects of different age groups. Click here for file
