# Supplementary material for: A longitudinal study of gene expression in healthy individuals
Source: BMC Med Genomics. 2009 Jun 7;2:33. doi: 10.1186/1755-8794-2-33 (PMC2713969; doi:10.1186/1755-8794-2-33)
Supplement: Additional file 1 — Primers used for qRT-PCR. The table provided describes the primer sequences used for qRT-PCR. [file 1755-8794-2-33-S1.doc]

## Additional file 1 – Primers used for qRT-PCR.

| **Genes** | **Genbank Accession Number*** | **Forward Primer** | **Reverse Primer** | **Amplicon Size^†^** |
| --- | --- | --- | --- | --- |
| **Target Genes** |  |  |  |  |
| CXCL1 | X54489 | CACCCCAAGAACATCCAAA | GCTCAAACACATTAGGCACAA | 292 |
| HMOX1 | CCDS13914.1 | TACCGCTCCCGCATGAA | GCTCCTGCAACTCCTCAA | 115 |
| ICAM1 | CCDS12231.1 | AACAACCGGAAGGTGTATGAA | CCTGGCAGCGTAGGGTAA | 190 |
| IL1B | CCDS2102.1 | CTGAGCTCGCCAGTGAA | GGTCTGTGGGCAGGGAA | 239 |
| IL1RN | CCDS2114.1 | CCGACCCTCTGGGAGAA | TCGCTCAGGTCAGTGATGTTAA | 270 |
| IL6R | CCDS1067.1 | CCCATCCCTGACGACAA | ACTGCTAACTGGCAGGAGAA | 123 |
| MMP9 | CCDS13390.1 | TCTGGAGGTTCGACGTGAA | AAGCGGTCCTGGCAGAAA | 139 |
| PTGS2 | CCDS1371.1 | AAGCTGGGAAGCCTTCTCTAA | CACAATCTCATTTGAATCAGGAA | 124 |
| SERPINE1 | CCDS5711.1 | GCGCTGCAGAAAGTGAA | TGTGCCGGACCACAAA | 136 |
| TGFB1 | CR601792, M38449 | CGTGCTCGCCCTGTACAA | GCTGAGGTATCGCCAGGAA | 322 |
| TNF | CCDS4702.1 | GCGGTGCTTGTTCCTCE | CATGGGCTACAGGCTTGT | 191 |
| **Reference Genes** | |  |  |  |
| 18S rRNA | X03205 | CGGGGAGGTAGTGACGAAAAATAE | CGGCTGCTGGCACCAGE | 121 |
| β2M | CCDS10113.1 | AACATGGAGACAGCACTCAAAGTAGAA | ACTGAATTCACCCCCACTGAAE | 282 |
| GAPDH | CCDS8549.1 | CGAGATCCCTCCAAAATCAA | CATGAGTCCTTCCACGATACCAA | 288 |
| PPP1CA | CCDS8160.1 | GGGCTTGAGGATCTGGAAA | GAGCACACCAGGTGGTAGAE | 158 |

^†^ The efficiency of each assay was between 93.0% (IL6R) and 105.9% (18S rRNA)

* The Genbank accession number listed is the NCBI reference sequence for the gene. More specifically, the consensus coding sequence is listed if it was available.

Note: E = t-butyl benzyl dA; X = t-butyl benzyl dC
