# Supplementary material for: A longitudinal study of gene expression in healthy individuals
Source: BMC Med Genomics. 2009 Jun 7;2:33. doi: 10.1186/1755-8794-2-33 (PMC2713969; doi:10.1186/1755-8794-2-33)
Supplement: Additional file 2 — Frequency distributions for white blood cell types for all subjects, all post-enrollment values. The histograms show distributions of cell counts for the major white blood cell types. [file 1755-8794-2-33-S2.doc]

## Additional file 2 – Frequency distributions for white blood cell types for all subjects, all post-enrollment values.


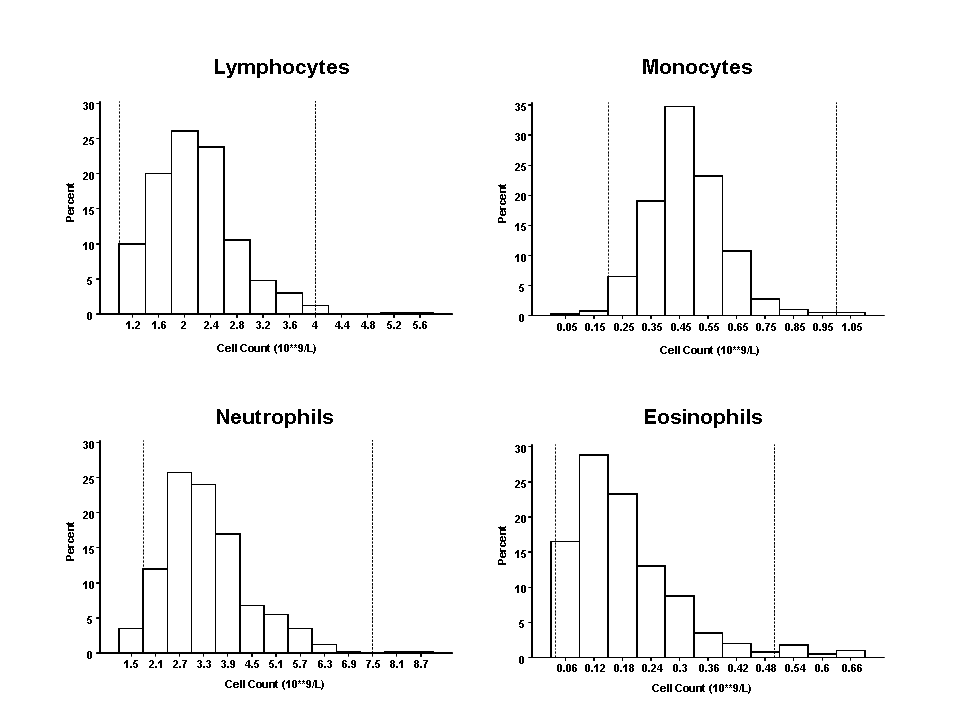


Dotted lines represent the upper and lower clinical reference values for the individual cell counts displayed.
