# Supplementary material for: A longitudinal study of gene expression in healthy individuals
Source: BMC Med Genomics. 2009 Jun 7;2:33. doi: 10.1186/1755-8794-2-33 (PMC2713969; doi:10.1186/1755-8794-2-33)
Supplement: Additional file 3 — Variability of raw and normalized Ct values (qRT-PCR study). The table shows the intra-individual variability in qRT-PCR Ct values for the subjects in the study. [file 1755-8794-2-33-S3.doc]

## Additional file 3 – Variability of raw and normalized C_t_ values (qRT-PCR study).

|  | **Mean** | **IQR** | **IQR** | **IQR** | **StdDev** | **StdDev** | **StdDev** |
| --- | --- | --- | --- | --- | --- | --- | --- |
|  | **Raw C_t_** | **Raw C_t_** | **Norm. C_t_** | **Diff.** | **Raw C_t_** | **Norm. C_t_** | **Diff.** |
| CXCL1 | 27.13 | 0.77 | 0.64 | 0.13 | 0.55 | 0.5 | 0.05 |
| HMOX1 | 25.87 | 0.86 | 0.73 | 0.13 | 0.59 | 0.55 | 0.04 |
| ICAM1 | 35.38 | 1.06 | 1.07 | -0.01 | 0.8 | 0.76 | 0.04 |
| IL1B | 25.52 | 0.68 | 0.64 | 0.03 | 0.56 | 0.51 | 0.05 |
| IL1RN | 25.28 | 0.79 | 0.53 | 0.26 | 0.61 | 0.56 | 0.05 |
| IL6R | 24.98 | 0.78 | 0.81 | -0.03 | 0.54 | 0.59 | -0.06 |
| MMP9 | 24.68 | 1.13 | 1.09 | 0.05 | 0.81 | 0.83 | -0.02 |
| PTGS2 | 26.95 | 0.97 | 0.91 | 0.07 | 0.60 | 0.55 | 0.04 |
| SERPINE1 | 30.40 | 1.20 | 1.47 | -0.26 | 0.91 | 0.96 | -0.06 |
| TGFB1 | 29.93 | 0.9 | 0.85 | 0.05 | 0.61 | 0.56 | 0.05 |
| TNF | 28.01 | 0.71 | 0.59 | 0.12 | 0.49 | 0.44 | 0.05 |

To evaluate the efficacy of reference gene normalization, C_t_ s were normalized to a weighted average of 4 reference genes (B2M, 18s rRNA, PPP1CA and GAPDH). IQR = inter-quartile range, the difference between the C_t_ of the 75^th^ and 25^th^ percentile. StdDev = standard deviation. Norm = normalized values, Raw = C_t_ value obtained after normalizing to total RNA input only, Diff = difference between raw and normalized C_t_ s.
