# Supplementary material for: A longitudinal study of gene expression in healthy individuals
Source: BMC Med Genomics. 2009 Jun 7;2:33. doi: 10.1186/1755-8794-2-33 (PMC2713969; doi:10.1186/1755-8794-2-33)
Supplement: Additional file 4 — Immunomodulatory genes differentially expressed by gender or age group (qRT-PCR study). The table shows variability in qRT-PCR Ct values between genders and subjects of different age groups. [file 1755-8794-2-33-S4.doc]

## Additional file 4 – Immunomodulatory genes differentially expressed by gender or age group (qRT-PCR study).

| **Gene** | **Females vs. Males**  **Fold Change** | ***p*-value** | **Old vs. Young Age Group Fold Change** | ***p*-value** |
| --- | --- | --- | --- | --- |
| CXCL1 | 1.31 | 0.029* | 1.09 | 0.428 |
| HMOX1 | 1.47 | 0.003* | 1.16 | 0.167 |
| ICAM1 | 1.42 | 0.036* | 1.19 | 0.153 |
| IL1B | 1.02 | 0.901 | 1.06 | 0.625 |
| IL6R | 1.11 | 0.302 | 1.07 | 0.252 |
| IL1RN | 0.95 | 0.699 | 1.22 | 0.110 |
| MMP9 | 1.17 | 0.316 | 1.27 | 0.035 |
| PTGS2 | 1.17 | 0.240 | 1.11 | 0.341 |
| SERPINE1 | 1.18 | 0.431 | 1.04 | 0.851 |
| TGFB1 | 1.05 | 0.680 | 1.08 | 0.494 |
| TNF | 1.15 | 0.200 | 0.92 | 0.376 |

Unadjusted p-values associated with each effect given. Fold changes were calculated as 2^β^, where β is the effect for gender or age group estimated from the mixed effects model for that gene. Fold changes estimated were the change in males with respect to female levels, and the change in older patients with respect to younger patients. The younger age group was defined as <= 55 years of age, while the older age group was defined as 56 years of age or more. Sample sizes for the groups were as follows: 8 young females, 7 older females, 5 young males, 8 older males.
